# Supplementary figures and images for: Particle swarm optimization solution for roll-off control in radiofrequency ablation of liver tumors: Optimal search for PID controller tuning
Source: PLoS One. 2024 Jun 26;19(6):e0300445. doi: 10.1371/journal.pone.0300445 (PMC11207125; doi:10.1371/journal.pone.0300445)

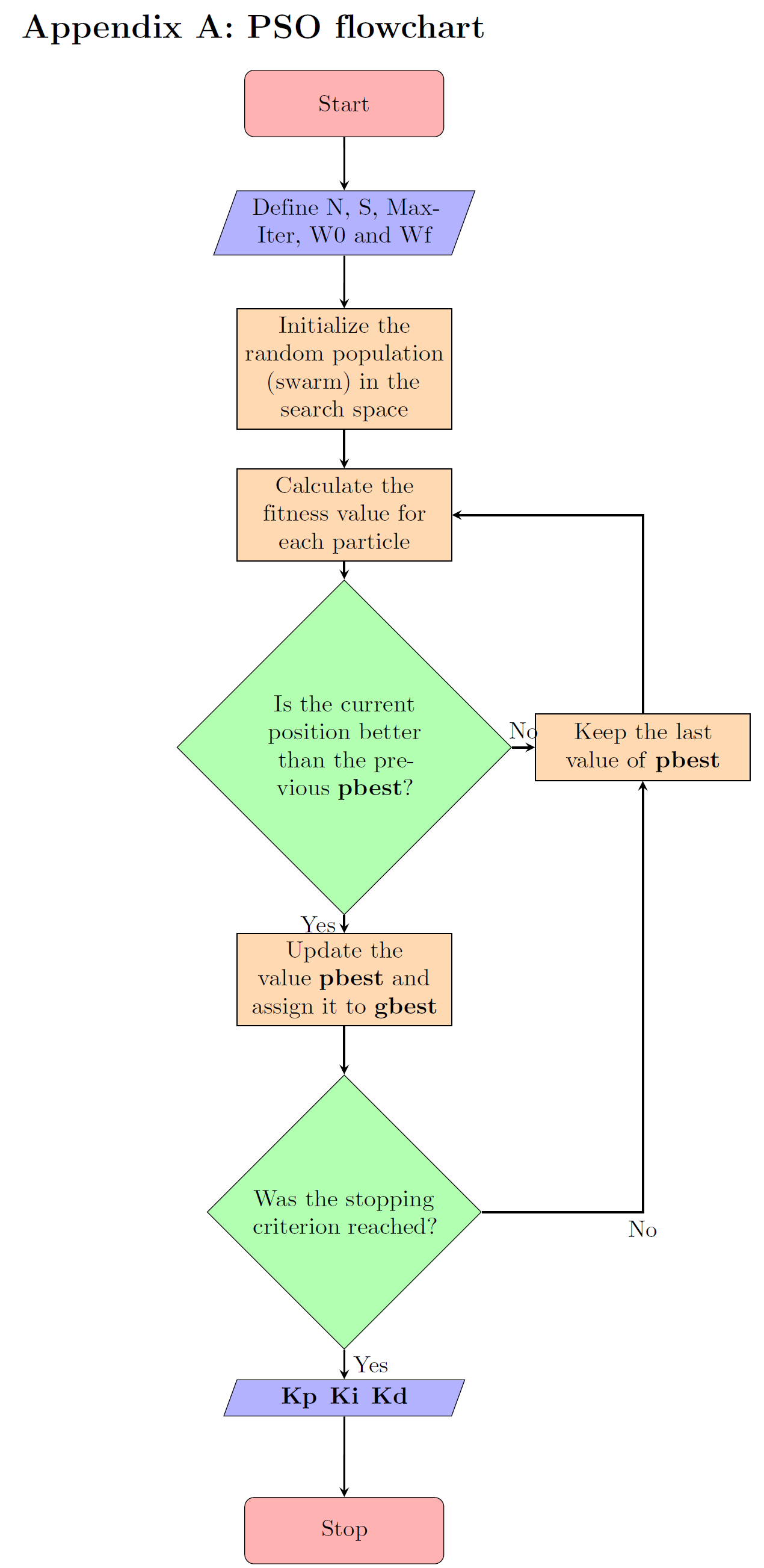

Supplement: S1 Fig — (TIF) [file pone.0300445.s001.tif]
