# Supplementary material for: Particle swarm optimization solution for roll-off control in radiofrequency ablation of liver tumors: Optimal search for PID controller tuning
Source: PLoS One. 2024 Jun 26;19(6):e0300445. doi: 10.1371/journal.pone.0300445 (PMC11207125; doi:10.1371/journal.pone.0300445)
Supplement: Attachment — Submitted filename: REBUTTAL LETTER - Rafael Mendes Faria.pdf [file pone.0300445.s006.pdf]

REBUTTAL LETTER

Dear Reviewers,

We extend our sincere gratitude for your thoughtful reviews and valuable insights into our manuscript. Your constructive feedback has been instrumental in refining our work. We have diligently endeavored to address each of your recommendations to the best of our ability, striving to enhance the clarity, rigor, and overall quality of the paper.

Your expertise and guidance have been immensely valuable in shaping the manuscript into a more robust and coherent piece of research. We appreciate the time and effort you dedicated to the evaluation of our work.

Should you have any further suggestions or if there are additional aspects that you deem pertinent for improvement, we are more than willing to accommodate them. Your feedback is crucial to the advancement of our research, and we are committed to ensuring that the final version of the manuscript meets the highest standards.

Review 01:

1) *In the title, the authors use the word "bio-inspired" to characterize what they do here. Are they making references to the fact that a bio-inspired optimization algorithm, say particle swarm optimization, is used? If so, please change the word "bio-inspired" with something like "particle swarm optimization", "optimum search", or similar. This would avoid any misunderstandings for the readers.*
Answer: Dear reviewer, thank you for this and all other suggestions regarding the manuscript submitted to the PLoS ONE journal. We will implement all recommendations to ensure our article fully meets the publication criteria set by PLoS ONE. Accordingly, we present the new title: "Particle Swarm Optimization Solution for Roll-off Control in Radiofrequency Ablation of Liver Tumors: Optimal Search for PID Controller Tuning."

2) *Among various optimization techniques, why didn't the authors use other methods like genetic algorithm (doi.org/10.1007/s11042-020-10139-6), bee colony algorithm (doi.org/10.1007/s10462-012-9328-0), and so on? These methods allow to have shorter computational times.*
Answer: First and foremost, I would like to express gratitude for your inquiry regarding the choice of optimization technique and for the suggestions and corrections proposed. It's important to acknowledge awareness of other optimization algorithms, such as the genetic algorithm (https://doi.org/10.1007/s11042-020-10139-6), the bee colony algorithm (https://doi.org/10.1007/s10462-012-9328-0), among others.
The decision to use Particle Swarm Optimization (PSO) was based on several considerations. Firstly, PSO demonstrated robust performance in estimating controller gains, resulting in performance indices such as overshoot, rise time, and steady-state error that met our quality criteria.
Furthermore, PSO was chosen due to its successful integration in other applications and research conducted by our research group. Specifically, we are implementing the PSO algorithm in the hardware of the SOFIA equipment, a crucial device for our studies. An

example of work with the SOFIA equipment can be found in our previous publication (https://doi.org/10.1109/TBME.2018.2873141).
It's essential to highlight that the current study under review is based on data obtained from the settings and results of this previous study. Therefore, the choice of PSO was motivated both by its reliable performance and our familiarity with its application in our research line.
I hope this information clarifies the selection of PSO as the optimization method in our article.
Once again, we appreciate your interest and contribution to improving our work.

3) *When writing the paper, it is suggested to avoid too many new lines since they make the text less fluente*.
Answer: Dear reviewer. We sincerely appreciate your valuable suggestions and the thorough review of our paper. We have carefully considered all your comments, especially regarding line breaks, and have taken the necessary steps to ensure the smooth flow of the text. We highly value your contribution to the quality of our work and are confident that the implemented changes will significantly enhance the clarity and readability of the article. Once again, we thank you for your time and effort devoted to reviewing our manuscript.

4) *When introducing the experimental protocol, are the authors sure that the experiments are design in order to avoid any heat losses to the environment? These losses might influence the ablated zone.*
Answer: Thank you for the pertinent observation regarding potential heat losses during our ex-vivo experiments. In our laboratory experiments, we implemented various measures to minimize heat dissipation to the surroundings. We carefully insulated the porcine liver samples using materials with low thermal conductivity, such as acrylic. This material was strategically positioned, limiting the samples' contact to a window of only 6 cm per side, which connected to the grounding surface. Additionally, we employed effective thermal insulation on the tray where the experiments were conducted. Furthermore, we maintained the laboratory temperature at approximately 22°C, relying on air conditioning systems to create an environment that replicated the same conditions for all conducted tests.

5) *In the model structure section, the authors report that the equipment used doesn't allow to measure at every constant time step. Is it reasonable to perform some linear interpolation? Please report some qualitative information about these time steps at which the equipment is able to perform measurements*.
Answer: The SOFIA equipment used in our ex-vivo experiments did not automatically export data at constant time intervals. Consequently, the collection of measurements, including impedance, voltage, current, and power, was done manually, leading to variations in the time intervals between each acquisition.
To address this variability in measurement time intervals, we utilized the "resample" command available in the MATLAB software. This command was employed to resample the non-uniform data and adjust it to a fixed rate using interpolation techniques. In the manuscript, we provide a more detailed and comprehensive description of how this tool was used to process the data and ensure the consistency of measurements over time.
We hope this information has clarified the procedure adopted to deal with variations in measurement time intervals. We appreciate you bringing this issue to our attention and trust that these explanations have been helpful.

6) *The authors claim that they use the ARX method to have some regressions between input and output (Eq. 1). Why didn't they use standard regressions or other AI techniques like for instance artificial neural networks?*

Answer: Dear reviewer, we appreciate your careful attention to our paper and the pertinent observation regarding the system identification method employed. We chose the AutoRegressive with eXogenous input (ARX) method to establish regressions between input and output (Eq. 1) based on considerations of simplicity and effectiveness for our specific objectives. The selection of the ARX method was motivated by its simplicity, aiding in result interpretation and understanding of the system's behavior. Additionally, ARX offers a transparent approach to modeling dynamic systems, which was crucial for our analysis.

As for not employing other techniques, such as artificial neural networks, it is a valid consideration. However, our primary focus was on simplicity and ease of incorporating the model into the SOFIA equipment, aiming for an efficient solution that is easily implementable in clinical practice. We believe that the chosen approach, combining ARX with PSO, provides a balanced solution for the specific objectives of our study.

7) *The reviewer thinks that the paper introduction is too much focused about roll-off phenomenon, even if in this field - tumor thermal ablation - other problems might arise and require several attention from the scientific community. These problems might be for instance tissue shinkrage (doi.org/10.1371/journal.pone.0210667) or underdesired healthy tissue damage (doi.org/10.3390/ma13010136), that might be treated with approaches based on customized procedures (doi.org/10.1016/j.cmpb.2020.105887) or different antennas to be used (doi.org/10.1080/10407782.2020.1835083). In order to underline the fact that optimizing radio-frequency ablation procedures - a widely used technique - still remains an open problem, the authors should consider all these aspects when writing their paper introduction.*

Answer: We appreciate the valuable guidance, which will undoubtedly enrich the context and relevance of our work in the scientific literature. We acknowledge the importance of broadening the focus beyond the roll-off phenomenon and addressing other critical challenges in the field of thermal tumor ablation. Apart from roll-off, there are crucial issues such as tissue shrinkage (doi.org/10.1371/journal.pone.0210667) and undesired damage to healthy tissues (doi.org/10.3390/ma13010136) that demand attention from the scientific community. Considering the complexity of these problems, we will emphasize in our introduction the need for a comprehensive approach to optimize radiofrequency ablation procedures. This will include exploring customized procedures (doi.org/10.1016/j.cmpb.2020.105887) and the potential use of different antennas (doi.org/10.1080/10407782.2020.1835083) to address these multifaceted issues. This broader approach will reflect the complexity of the clinical scenario and the ongoing quest for effective solutions in thermal ablation, thereby contributing to a more holistic understanding of the field.

8) *When validating the regression model, it is suggested to use some graphs to show that the coefficient of determination (Eq. 6) is not that high; a graph that report experimental outcomes (x-axis) and regressed model (y-axis) with the bisector could be an idea.*

Answer: We appreciate the valuable suggestion. The requested graph has already been incorporated into Figure 5 of the manuscript. In this representation, it is possible to visualize the relationship between the experimental results and the fitted model. It is essential to highlight that, due to the high coefficient of determination, the measured data and those obtained in the regression are practically overlapped, indicating good agreement. To ensure full transparency, all calculations used in this analysis are thoroughly described in the ARX

code, available as part of the supplementary material..

9) *When describing the particle swarm optimization algorithm, even if the text is quite clear, it is suggested to show a figure/table that clearly presents input variables (to be changed), constraints (to be not modified) and objective function; the authors could also integrate this within Fig. 1.*
Answer: We greatly appreciate the valuable suggestion for improving our article. In response to your recommendation, we have added an additional block to Figure 1, and its corresponding expansion is now highlighted in Figure 2, clearly presenting all input variables (to be changed), constraints (not to be modified), and the objective function. We hope that these changes significantly enhance the understanding of the PSO algorithm, providing a more comprehensive and elucidative visual representation..

10) *When the optimum solution is obtained, it is suggested to report a comparison between the optimum solution and a reference solution, to appreciate how important is to run optimization for this specific case.*
Answer: We appreciate the reviewer's suggestion, and we have included a detailed comparison between the obtained optimum solution and a reference solution in the manuscript. This comparison provides insights into the significance of running the optimization for this specific case, as suggested. The relevant information and analysis can be found in discussion of the revised manuscript.

11) *In the manuscript, with particular references to abstract and discussion sections, it is suggested to provide more practical outcomes from the present works, like for instance more details about the optimum procedure that has been found. This would be really helpful in medical practice.*
Answer: Dear Reviewers, we wish to express our sincere appreciation for the invaluable feedback and constructive comments you provided in your evaluation of the manuscript. Your wealth of experience and insightful guidance are of immense importance to us, and we hold your expertise in the highest regard. We extend our gratitude for the time and meticulous attention you dedicated to proofreading; we deeply acknowledge the significance of your thorough review.
Regarding your request, we wholeheartedly understand the importance of presenting more practical results, especially within the summary and discussion sections of our article. Your suggestion to furnish additional details on the optimal procedure revealed by our research aligns seamlessly with our commitment to enhancing the clinical applicability of our work.
In response, we have implemented the following adjustments to the Abstract:

- Rewritten the Abstract to enhance clarity and conciseness.
- Emphasized the potential to reduce incomplete tumor ablation, improve patient outcomes, and diminish tumor recurrence rates.
- Clarified the objective statement to facilitate better comprehension.

In response, we have implemented the following adjustments to the Discussion:

In the discussion, adjustments were made to emphasize the clinical significance of the study, provide a detailed description of the optimized procedure, highlight safety and cost considerations, underscore the need for training and comprehensive adoption, compare the new approach to existing methods, and emphasize the need for future research. The discussion

concluded with comments on the study's potential and next steps.

12) *As a potential future work, the authors could also think about considering a different objective function, like for instance the ablation zone with no necrosis for the healthy tissue. Another idea could be to run a multi-objective optimization analysis considering also aspects like exposure time.*
Answer: We want to express our sincere gratitude to the reviewers for their invaluable insights and innovative suggestions. We are committed to incorporating objective function analysis, especially focused on protecting healthy tissue and eliminating satellite tumors in our future work. Your insights have significantly enriched our research and opened new possibilities to enhance the outcomes of our ablation procedures. Once again, we thank you for your invaluable contribution.
We've added 3 more paragraphs to the topic to meet the reviewers' demands.

Review 02:

1. *The abstract is very lengthy. Rewrite it in a concise manner and mention in it the obtained findings and show some statistical numbers.*
   Answer: Thank you for your valuable feedback. We have revised the abstract to enhance conciseness while highlighting key findings and incorporating statistical data. The updated abstract now provides a succinct overview of the study, including significant quantitative results, contributing to a more focused and informative presentation.

2. *The manuscript needs a comprehensive English language proofreading, there are many linguistic mistakes.*
   Answer: Thank you for the observation. We have enlisted the services of the university's English correction service to enhance the linguistic quality of the manuscript. However, we acknowledge the importance of linguistic accuracy, and if necessary, we are open to considering the hiring of PLOS ONE's revision services to ensure excellence in the article's presentation. We are committed to ensuring that the manuscript meets the required quality standards.

3. *The introduction does not provide sufficient background information for readers not in the immediate field to understand the problem/hypotheses.*
   Answer: We sincerely thank you for your valuable observations and comments. We have taken your suggestions on board and, based on them, carried out a comprehensive revision of the article's introduction. We have made significant changes, including rewriting paragraphs and adding more references to provide a more solid foundation for readers who may not be familiar with the field.
   Our main intention was to more clearly highlight the problem at hand and the underlying hypotheses, making the text more accessible and understandable. We hope that the changes made have met these objectives, and we look forward to receiving further guidance should there be a need for additional improvements.
   Thank you again for your time and effort in reviewing our article.

4. *The literature survey is not balanced, important studies are not cited in this work, the following papers are very close to the topic of the paper and must be cited,*

- *doi:10.1177/1729881420981524*
- *https://doi.org/10.1049/tje2.12009*
- *https://doi.org/10.1016/B978-0-12-820276-0.00026-1*
- *doi.org/10.1177/0020294020917171*
- https://doi.org/10.1155/2020/3067024

  Answer: Thank you for the careful observation and the indication of relevant references. We appreciate the importance of a comprehensive literature review. We will carefully analyze the suggested articles and ensure the proper inclusion of those directly related to the topic of our work. This will enrich the theoretical foundation of the article, providing a more complete and accurate understanding of the context of our study.

5. *I think the motivations for this study need to be made clearer.*
Answer: The motivation underlying this study lies in the quest to enhance the effectiveness of radiofrequency ablation (RFA) in the treatment of hepatic lesions, with a specific focus on the roll-off phenomenon. This phenomenon, resulting in the premature reduction of thermal energy and thereby limiting the treatment's efficacy, poses a significant challenge. The use of the proportional-integral-derivative (PID) controller emerges as a key strategy to mitigate the negative effects of roll-off, allowing for more precise dynamic control of temperature during the RFA procedure. The implementation of swarm-based optimization techniques, such as the PSO algorithm, to fine-tune PID parameters aims to optimize system performance. The study thus aspires to contribute to the understanding and mitigation of roll-off, offering an innovative and effective approach to improve clinical outcomes in radiofrequency ablation. Dear reviewer, this section related to the motivation is found in the last 5 paragraphs of the introduction, under the subtopic “Roll-off and Coagulation Zone”.

6. *The study objectives must be clearly defined.*
Answer: We appreciate the reviewer's feedback. In response to the suggestion, we have carefully revised and clearly defined the study objectives in the introduction section of the manuscript. The updated version now explicitly outlines the goals and aims of the research to provide a better understanding for the readers. We believe that these changes enhance the overall clarity and focus of the study.

7. *No comparisons found with recent works, these are necessary to verify the effectiveness of the proposed designs.*
Answer: We would like to express our gratitude to the reviewer for the valuable feedback. In response to the suggestion, we have conducted a thorough review of recent works in the field and incorporated relevant comparisons in the manuscript. The updated version now includes a comparative analysis with recent studies to provide a comprehensive understanding of the effectiveness of the proposed designs. We believe that these additions contribute significantly to the overall robustness and context of our research.

8. *The results are not clearly explained and must be presented in an appropriate format.*
Answer: Dear reviewer, the discussion has been restructured once again, following the guidelines from the PLOS ONE author guide. As per the recommendations, we aimed to encompass the results of our research, a discussion of related studies, and a comparison between our findings and the initial hypothesis.

9. *Stability analysis of the closed-loop system in the presence of the proposed PSO is not available, it must be addressed.*
Answer: Thank you for the relevant observation. Based on your suggestion, we conducted a closed-loop stability analysis using the Routh-Hurwitz method, and the results have been incorporated into Table 4 of the article. This inclusion aims to provide a comprehensive assessment of the system's stability with the implementation of the proposed PSO. If there are additional points that deserve attention or if further information is needed, we are open to more suggestions to enhance the quality of the work.

10. *The findings are not properly described in the context of the published literature.*
Answer: We appreciate the reviewer's feedback and acknowledge the importance of contextualizing our findings within the existing literature. In response to this suggestion, we have revisited the manuscript and provided a more detailed discussion that explicitly connects our findings with relevant published literature. This clarification aims to enhance the coherence of the paper and ensure a more comprehensive understanding of how our results contribute to the current state of knowledge in the field. We believe that these improvements address the reviewer's concern and strengthen the overall quality of the manuscript.

11. *No significant limitations are discussed. It may be worthwhile to mention the tradeoffs involved in your work.*
Answer: Thank you for your valuable feedback. We appreciate the suggestion to discuss the limitations and tradeoffs of our work more explicitly. In response to this, we have revised the manuscript to include a dedicated section discussing the limitations and tradeoffs associated with our approach. By highlighting these aspects, we aim to provide a more thorough and balanced perspective on the scope and applicability of our study. We trust that this addition enhances the overall quality of the manuscript and provides a more comprehensive understanding for readers.

Reviewer 3:

1. *Results shown can be shown in a tabular form.*
Answer: Thank you for your observation. We have taken note of your suggestion. In the revised version of the manuscript, we will present the results in a tabular form to facilitate a clearer and more organized presentation of the findings. This tabular representation will offer readers a concise and structured view of the results, enhancing the overall accessibility and interpretation of the data. We appreciate your guidance and believe that this modification will contribute to the clarity and effectiveness of our manuscript.

2. *Some recent works should be referred in literature and in work.*
Answer: Agradecemos pela observação valiosa. Conforme sua sugestão, revisamos e incorporamos recentes trabalhos relevantes à literatura no nosso manuscrito. Essas referências adicionais fortalecerão a fundamentação teórica e a conexão do nosso trabalho com as pesquisas mais recentes na área. Caso haja alguma sugestão específica ou se desejar mais detalhes sobre as referências incluídas, ficamos à disposição para fornecer informações adicionais.

3. *Graphical analysis should be presented.*
   Answer: Thank you for your valuable suggestion. We acknowledge the importance of graphical analysis for a clearer presentation of the data and findings. In response to your feedback, we will enhance the manuscript by incorporating graphical representations to illustrate key points, trends, and comparisons. These visual aids will provide readers with a more comprehensive and intuitive understanding of the results. We appreciate your guidance, and this improvement will contribute to the overall quality and accessibility of our research.

4. *Conclusion should include quantitative results.*
   Answer: Thank you for your suggestion. We have revised the conclusion to incorporate quantitative results, specifically citing performance indices such as overshoot, rise time, and settling time. This addition enhances the conclusion by providing a more specific and measurable summary of the study's outcomes.
