# Supplementary material for: Particle swarm optimization solution for roll-off control in radiofrequency ablation of liver tumors: Optimal search for PID controller tuning
Source: PLoS One. 2024 Jun 26;19(6):e0300445. doi: 10.1371/journal.pone.0300445 (PMC11207125; doi:10.1371/journal.pone.0300445)
Supplement: Attachment — Submitted filename: Response to Reviewers.pdf [file pone.0300445.s007.pdf]

REBUTTAL LETTER

Dear Reviewers,

We extend our sincere gratitude for your thoughtful reviews and valuable insights into our manuscript. Your constructive feedback has been instrumental in refining our work. We have diligently endeavored to address each of your recommendations to the best of our ability, striving to enhance the clarity, rigor, and overall quality of the paper.

Your expertise and guidance have been immensely valuable in shaping the manuscript into a more robust and coherent piece of research. We appreciate the time and effort you dedicated to the evaluation of our work.

Review 04:

1) *Please improve figures quality.* OK
Answer: Dear Reviewer,
I want to express my gratitude for all the considerations made regarding our work. I am confident that by following your suggestions, our work will reach a level of excellence that it would not have without them. The figures have been enhanced in quality following the guidelines of PLoS ONE. The graphs were extracted from MATLAB software, exported with the best available resolution, and verified for dimensions and quality using the PACE platform (PLoS ONE).

2) *Please add proper flowchart.* OK
Answer: Dear Reviewer,
I would like to thank you once again for this valuable contribution to improving our work. The flowchart has been appropriately added at the end of the paper in Appendix A, and it is also included in the supplementary materials, as explained at the end of the Materials and Methods section.

3) *Please add block diagram.* OK
Answer: Dear Reviewer,
The block diagram has been inserted in place of Figure 4. The block diagram highlights the circuit designed in Simulink software for determining the gains Kp, Ki, and Kd of the PID controller. Additionally, it shows the presence of the ISE and ISTE errors, which are calculated and sent to the script to compose the objective function.

4) *Please justify gap and its addressing by your paper in literature.* OK
Answer: Dear Reviewer,
Thank you for your insightful questions regarding our article. Your inquiries are elevating the quality of our study. We have added a paragraph at the end of the introduction to justify the gap in the literature that our study addresses. We focus on the lack of investigations into roll-off displacement with precise automatic adjustment control using bioinspired computational techniques such as PSO. Additionally, it aims to enable deployment of this control on hardware for real-time execution. In future work, we plan to integrate a dynamic actuation controller. This controller will receive information on tissue impedance variation and simultaneously adjust the PID controller to displace the roll-off, thus providing a more efficient ablation procedure.

Review 05:

It is a detailed theoretical and experimental study and the mostly the reviewers' comments are responded to; however, still, the following comments should be addressed:

1) *Careful attention should be given to correcting writing mistakes.* OK
Answer: Dear Reviewer,
Thank you for your feedback. We have carefully reviewed the paper and made the necessary corrections to address writing mistakes. Your input has been invaluable in improving the quality of our manuscript.

2) *Although the introduction and related work have been presented, the differences of the study should have been briefly highlighted in related work.* OK
Answer: Dear Reviewer,
Thank you for your consideration and suggestion regarding the topic in question. A paragraph has been added to the "Related Work" section briefly describing the differences in the study, as requested.

3) *I recommend referencing the following publication for PSO and PID techniques: https://doi.org/10.1007/978-3-031-26876-2_85 . Additionally, more recent references from recent years should be included.* OK
Answer: Dear Reviewer,
We greatly appreciate your suggestion. The study *https://doi.org/10.1007/978-3-031-26876-2_85* has been included in the "Related Works" section as requested. Additionally, throughout the text, new references have been added to strengthen and substantiate our study in the literature.

4) *In Fig 4, the peak point of the voltage, the stabil voltage, and applied voltage have not been explained in detail. The Voltage stabil time and later peak voltage should be explined.* OK
Answer: Dear Reviewer,
Thank you for your valuable feedback. Regarding Figure 4, which depicts the voltage characteristics during our experiments, we have revised the text to provide a more detailed explanation of the peak point of the voltage, the stabilized voltage, and the applied voltage. We have also included an explanation of the voltage stabilization time and the later peak voltage. These changes aim to enhance the clarity and completeness of our presentation of the experimental data.

5) *The meaning of the map and numbers in the root locus should be explained more thoroughly.* OK
Answer: Dear Reviewer,
Thank you for your feedback. We have revised the explanation of the root locus diagram to provide a more thorough understanding of its meaning and the significance of the numbers displayed.

6) *Overall ,except Figures 1 and 2, there is lack of detail in explanation for all the figures.* *OK*
<u>Answer:</u> Dear Reviewer,
Thank you for your feedback regarding the figure captions. We have added information to the captions and improved how the information was conveyed. Additionally, we have enhanced the quality of all figures and included a new Figure 4, which caused all other figures to be renumbered accordingly.

Review 06:

1) *The references provided are all applicable but not sufficient because there are some parts in the paper that need to be cited appropriately.* *OK*
<u>Answer:</u> Dear Reviewer,
Thank you for your feedback. We have carefully reviewed the paper and added several references to address the points that needed to be cited appropriately. We believe these additions have significantly improved the paper's completeness and relevance to the existing literature.
